# Application of artificial intelligence in chronic liver diseases: a systematic review and meta-analysis

**DOI:** 10.1186/s12876-020-01585-5

**Published:** 2021-01-06

**Authors:** Pakanat Decharatanachart, Roongruedee Chaiteerakij, Thodsawit Tiyarattanachai, Sombat Treeprasertsuk

**Affiliations:** 1 Department of Medicine, Faculty of Medicine, Chulalongkorn University, Bangkok, Thailand; 2 Division of Gastroenterology, Department of Medicine, Faculty of Medicine, Chulalongkorn University and King Chulalongkorn Memorial Hospital, Thai Red Cross Society, 1873 Rama IV Rd., Pathum Wan, Bangkok, 10330 Thailand; 3 Center of Excellence for Innovation and Endoscopy in Gastrointestinal Oncology, Faculty of Medicine, Chulalongkorn University, Bangkok, Thailand; 4 Faculty of Medicine, Chulalongkorn University, Bangkok, Thailand

**Keywords:** Artificial intelligence, Computer-assisted, Machine learning, Deep learning, Liver fibrosis, Cirrhosis, Liver steatosis, Fatty liver, NAFLD, Non-invasive diagnostic tests

## Abstract

**Background:**

The gold standard for the diagnosis of liver fibrosis and nonalcoholic fatty liver disease (NAFLD) is liver biopsy. Various noninvasive modalities, e.g., ultrasonography, elastography and clinical predictive scores, have been used as alternatives to liver biopsy, with limited performance. Recently, artificial intelligence (AI) models have been developed and integrated into noninvasive diagnostic tools to improve their performance.

**Methods:**

We systematically searched for studies on AI-assisted diagnosis of liver fibrosis and NAFLD on MEDLINE, Scopus, Web of Science and Google Scholar. The pooled sensitivity, specificity, positive predictive value (PPV), negative predictive value (NPV) and diagnostic odds ratio (DOR) with their 95% confidence intervals (95% CIs) were calculated using a random effects model. A summary receiver operating characteristic curve and the area under the curve was generated to determine the diagnostic accuracy of the AI-assisted system. Subgroup analyses by diagnostic modalities, population and AI classifiers were performed.

**Results:**

We included 19 studies reporting the performances of AI-assisted ultrasonography, elastrography, computed tomography, magnetic resonance imaging and clinical parameters for the diagnosis of liver fibrosis and steatosis. For the diagnosis of liver fibrosis, the pooled sensitivity, specificity, PPV, NPV and DOR were 0.78 (0.71–0.85), 0.89 (0.81–0.94), 0.72 (0.58–0.83), 0.92 (0.88–0.94) and 31.58 (11.84–84.25), respectively, for cirrhosis; 0.86 (0.80–0.90), 0.87 (0.80–0.92), 0.85 (0.75–0.91), 0.88 (0.82–0.92) and 37.79 (16.01–89.19), respectively; for advanced fibrosis; and 0.86 (0.78–0.92), 0.81 (0.77–0.84), 0.88 (0.80–0.93), 0.77 (0.58–0.89) and 26.79 (14.47–49.62), respectively, for significant fibrosis. Subgroup analyses showed significant differences in performance for the diagnosis of fibrosis among different modalities. The pooled sensitivity, specificity, PPV, NPV and DOR were 0.97 (0.76–1.00), 0.91 (0.78–0.97), 0.95 (0.87–0.98), 0.93 (0.80–0.98) and 191.52 (38.82–944.81), respectively, for the diagnosis of liver steatosis.

**Conclusions:**

AI-assisted systems have promising potential for the diagnosis of liver fibrosis and NAFLD. Validations of their performances are warranted before implementing these AI-assisted systems in clinical practice.

*Trial registration*: The protocol was registered with PROSPERO (CRD42020183295).

## Background

Chronic liver diseases and cirrhosis are the 11th leading cause of death in the world, accounting for 1.1 million deaths annually [1]. The global prevalence of cirrhosis has been substantially rising from 71 million in 1990 to over 122 million in 2017 [2]. Common causes of cirrhosis are chronic hepatitis B virus (HBV) and hepatitis C virus (HCV) infections, alcohol-related liver disease and nonalcoholic steatohepatitis (NASH) [2]. Over the past decade, there has been a temporal shift in the prevalence of causes of cirrhosis, i.e., the prevalence of NASH has been dramatically increasing, whereas the prevalence of other causes has been slowly decreasing [3]. The estimated worldwide prevalence of nonalcoholic fatty liver disease (NAFLD) is 25% [4] and is projected to be to 33.5% by 2030, emphasizing the importance of both cirrhosis and NAFLD [5].

The spectrum of liver fibrosis ranges from minimal fibrosis to full-blown cirrhosis [6]. Patients with early cirrhosis are mostly asymptomatic because the liver is able to compensate. However, without a prompt diagnosis and proper treatments, it can quickly deteriorate to decompensated cirrhosis, which eventually leads to complications and mortality. Patients with decompensated cirrhosis have an approximately tenfold higher risk of death than general populations [7]. Therefore, the detection and treatment of early-stage fibrosis and NASH can slow disease progression, reduce the risk of liver cancer and decrease mortality.

The gold standard for the diagnosis and staging of liver fibrosis and NAFLD is liver biopsy. However, liver biopsy is an invasive procedure that can lead to complications such as hemorrhage, biliary peritonitis and pneumothorax [8]. Another drawback of liver biopsy is a high rate of sampling error with interobserver and intraobserver variation in histologic evaluations [6, 9]. Additionally, liver biopsy is not always feasible as a follow-up method for liver diseases. Accordingly, serum markers and imaging modalities have been developed as alternative noninvasive diagnostic methods for liver fibrosis, but they have limited performance, particularly for early-stage fibrosis [8, 10]. For example, the sensitivity and specificity of the aspartate aminotransferase-to-platelet ratio index (APRI) are 69% and 77%, respectively, and those of the Fibrosis-4 (FIB-4) score are 69% and 78%, respectively, for the detection of advanced fibrosis [11]. Various imaging modalities, e.g., magnetic resonance elastography (MRE), have also been used for the diagnosis and classification of liver fibrosis with relatively reliable accuracy [12]. However, the availability of these modalities is limited. The performance of most of these tests needs to be improved.

Since the twenty-first century, there have been significant advancements in artificial intelligence (AI) technology, resulting in applications of AI in several aspects of medicine, particularly in aiding diagnosis. In gastroenterology, AI-assisted systems have been studied in various diseases such as the endoscopic detection and classification of colorectal cancer [13, 14]. Regarding the application of AI in liver diseases, machine learning algorithms has been developed to predict risk and outcomes of diseases using multiple clinical parameters, e.g. assessment of liver fibrosis and steatosis, predicting liver decompensation in primary sclerosing cholangitis, screening and selection of liver transplant recipients as well as predicting post-transplant survival and complications [15].

There have been some previous systematic reviews on AI in gastroenterology and liver disease [15, 16], however, very few meta-analyses have been conducted to evaluate the performance of the AI-assisted systems. In this systematic review and meta-analysis, we focused mainly on liver parenchymal diseases, i.e., liver fibrosis and steatosis. The main objective of this study was to assess the performance of AI-integrated noninvasive tests for the diagnosis and staging of liver fibrosis and steatosis.

## Methods

The study was conducted based on the Preferred Reporting Items for Systematic Review and Meta-Analysis (PRISMA) checklist.

### Search strategy

We searched for studies on AI in liver fibrosis and steatosis. A literature search was conducted on MEDLINE, Scopus, Web of Science and Google Scholar databases. The search was conducted from the year 2000 through January 2020. We opted to exclude studies published before 2000 because most of these studies utilized obsolete computer-assisted algorithms that are currently no longer used in the modern AI era. Keywords for the search were as follows: “artificial intelligence”, “computer-assisted”, “computer-aided”, “neural network”, “machine learning”, “deep learning”, “liver”, “hepatic”, “parenchyma”, “parenchymal”, “fibrosis”, “cirrhosis”, “steatosis”, “fatty”, “NASH”, and “NAFLD”.

### Inclusion and exclusion criteria

We included all articles focusing on the utilization of AI in the diagnosis and/or staging of liver fibrosis and steatosis. The inclusion criteria were as follows: participants included in the study underwent liver biopsy as the gold standard for the diagnosis of liver fibrosis and steatosis. The reported results were sufficient for generating 2 × 2 tables, and the articles were in English. The exclusion criteria were as follows: articles that did not report our desired outcomes of sensitivity, specificity, positive predictive value (PPV), negative predictive value (NPV); studies that did not provide sufficient information to calculate true positive (TP), false positive (FP), true negative (TN) and false negative (FN) values; articles that did not clearly report training and test datasets or did not contain information on validation methods; and conference proceedings or abstracts with incomplete information on population, AI methods, and validation methods.

### Data extraction and quality assessment

Two authors (PD and TT) independently performed data extraction and quality assessment. Any disagreements were discussed with the third author (RC). Data extracted included the author, publication year, country where the study was conducted, study design, liver diseases/conditions, diagnostic modalities, number of participants, type of AI models, number of samples in the development and validation cohorts, validation method (e.g., k-fold cross validation, independent cohort), sensitivity, specificity, and crude number of TP, FP, TN and FN values. For the studies that developed multiple AI models, we included the AI model that had the best overall performance in the main analysis. Our criterion for the best overall performance was to calculate the mean between the sensitivity and specificity, i.e., (sensitivity + specificity)/2 [17]. This criterion was used because we equally emphasized the sensitivity and specificity. In the diagnosis of liver fibrosis, especially cirrhosis, we would like a diagnostic test to be sensitive in order to early detect liver fibrosis. However, we would also like to avoid incorrectly diagnosing patients as having liver fibrosis when they actually do not have the condition. Therefore, we opted for methods with a balanced false negative (sensitivity) and false positive (specificity) [17]. Moreover, sensitivity and specificity do not depend on prevalence or incidence in validation cohorts. We also extracted performance of AIs with the best sensitivity and specificity in studies with multiple AIs models in order to further perform sensitivity-focused and specificity-focused analysis.

### Quality assessment

The methodological quality of the included studies was evaluated using the Quality Assessment of Diagnostic Accuracy Studies (QUADAS-2) tool [18]. The QUADAS-2 tool comprises 12 questions regarding 4 domains including patient selection, index test, reference standard, and flow and timing. Some questions were slightly modified to specifically assess studies on AI. For example, in clinical studies on diagnostic tests, prespecified thresholds of the index test should be set prior to data collection and analysis to prevent post-hoc data analysis for the desired results. For AI research, we assessed this issue by identifying whether the developed AI model was validated in another set of cohorts apart from the training cohorts, e.g., test set, or external validation cohorts. Details of the modified QUADAS-2 tool are provided in the Supplemental methods.

### Statistical analysis

After data extraction, the TP, FP, TN and FN values, if not available, were calculated using Review Manager version 5.3.5 [19]. All statistical analyses were performed using R software, version 3.6.3, Vienna, Austria [20]. The pooled sensitivity, specificity, positive predictive value (PPV), negative predictive value (NPV) and diagnostic odds ratio (DOR) with 95% confidence intervals (95% CIs) were calculated from the crude number of TP, FP, TN and FN values of each study using a random effects model. The summary receiver operating characteristics (SROC) curve was generated, and the area under the curve (AUC) was calculated to determine the diagnostic accuracy of the AI-assisted system. AUC values of 0.5–0.7, 0.7–0.9, and 0.9–1 indicate low, moderate and high accuracy, respectively [21]. Heterogeneity was assessed using *I*^2^ and Cochran’s Q statistics. To determine the source of heterogeneity, subgroup analyses and regression analysis based on diagnostic modalities, population and AI classifiers were performed. Publication bias was assessed with the Deeks funnel plot. *P* values of < 0.05 were considered statistically significant.

## Results

### Literature search

**Fig. 1:**
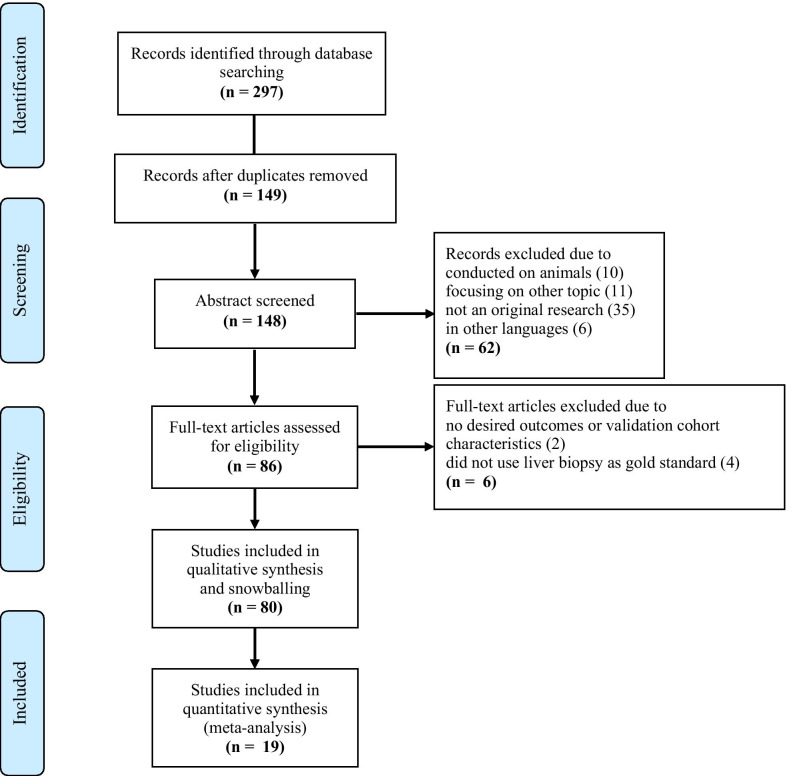
Flow diagram of search methodology and literature selection process

The search results and process of selecting articles are shown in Fig. 1. After the literature search, a total of 297 articles were identified. Articles were excluded for the following reasons: studies that were duplicated (n = 149), studies that were conducted in animals (n = 10), studies focusing on diseases other than liver parenchymal diseases (n = 11), studies that were not original research, i.e., reviews, editorials (n = 35), studies that were not written in English language (n = 6), studies that did not report the desired outcomes or validation population characteristics (n = 2), and studies that did not use liver biopsy as the gold standard (n = 4). Eventually, a total of 80 articles were included in the qualitative analysis and snowballing, of which 19 were included in the quantitative analysis (17 studies on liver fibrosis and 2 studies on NAFLD). There were 12 studies integrating AI with imaging modalities, i.e., ultrasonography [22–26], elastography [27, 28], computed tomography (CT) [29, 30] and magnetic resonance imaging (MRI) [31, 32], to facilitate the diagnosis of liver fibrosis and NAFLD. The other 7 studies developed AI models using clinical and laboratory data, such as the presence of other underlying diseases or ascites, liver chemistry tests, and platelet and white blood cell counts, to predict liver fibrosis stages [33–39]. Regarding the types of AI, 6 studies used convolutional neural networks (CNNs) [22, 24, 28–30, 32], 6 studies used artificial neural networks (ANNs) [25, 26, 35–37, 39], 5 studies used multiple AI models [23, 27, 33, 34, 38] and 2 studies used a support vector machine (SVM) [31, 40]. The study characteristics, sensitivity, specificity, prevalence, validation methods and other extracted data from the included studies are shown in Table 1. The methodological assessment by QUADAS-2 is summarized in Additional file 1: Table S1.

**Table 1:** Characteristics of included studies

Study/year	Country	Study cohort	Population	AI classifier	AI modality	Development cohort	Validation cohort	Validation methods	Stage	Sensitivity	Specificity	TP	FP	TN	FN
Zhang, 2012 [25]	China	Prospective	Chronic hepatitis B, C	ANN	USG	F1/F2/F3/F4 40/22/55/62	F1/F2/F3/F4 13/8/19/20	Independent test set	F4	0.95	0.85	19	6	34	1
Chen, 2017 [27]	China	Prospective	Chronic hepatitis B	NB^a^, RF^a^, KNN, SVM	Elastography	S0/S1/S2/S3/S4 119/164/88/72/70	N/A	k-fold cross validation	F4	0.6866	0.8854	48	51	392	22
									≥ F3	0.7866	0.8738	112	47	324	30
									≥ F2	0.7471	0.8621	172	39	244	58
									≥ F1	0.7967	0.8250	314	21	98	80
Choi, 2018 [29]	Korea	Retrospective	General population	CNN	CT	F0/F1/F2/F3/F4 3357/113/284/460/3247	F0/F1/F2/F3/F4 118/109/161/173/330	Independent test set	F4	0.846	0.966	279	19	242	51
									≥ F3	0.946	0.954	476	18	370	27
									≥ F2	0.955	0.899	634	23	204	30
Yasaka, 2018 [30]	Japan	Retrospective	General population	CNN	CT	F0/F1/F2/F3/F4 113/36/56/66/125	F0/F1/F2/F3/F4 29/9/14/16/32	Independent test set	F4	0.75	0.57	24	29	39	8
									≥ F3	0.75	0.65	36	18	34	12
									≥ F2	0.76	0.68	47	12	26	15
Yasaka, 2018 [32]	Japan	Retrospective	General population	CNN	MRI	F0/F1/F2/F3/F4 54/53/81/113/233	F0/F1/F2/F3/F4 10/10/15/20/45	Independent test set	F4	0.76	0.76	34	13	42	11
									≥ F3	0.78	0.74	51	9	26	14
									≥ F2	0.84	0.65	76	3	7	14
Li, 2019 [23]	China	Prospective	Chronic hepatitis B	Adaboost^a^, DT, LR, ANN, RF, SVM	USG	F0/F1/F2/F3/F4 15/33/38/23/35	N/A	Tenfold cross validation	≥ F2	0.875	0.769	84	11	37	12
Wang, 2019 [28]	China	Prospective	Chronic hepatitis B	CNN	Elastography	F0-1/F2/F3/F4 43/72/85/66	F0-1/F2/F3/F4 22/37/41/32	Independent test set	F4	0.969	0.88	31	12	88	1
									≥ F3	0.904	0.983	66	1	58	7
									≥ F2	0.691	0.909	76	2	20	34
Ahmed, 2020 [31]	Egypt	Prospective	Chronic hepatitis C	SVM	MRI	22 fibrotic patients 15 healthy patients	N/A	Leave one out cross validation	≥ F1	0.818	0.866	18	2	13	4
Lee, 2020 [22]	Korea	Retrospective	Chronic liver disease, hepatitis B, C	CNN	USG	F0/F1/F23/F4 363/394/1652/1566	F0/F1/F23/F4 290/17/72/193	Independent test set	F4	0.778	0.937	150	24	355	43
									≥ F2	0.913	0.824	242	54	253	23
Schawkat, 2020 [40]	Switzerland	Prospective	General population	SVM	MRI	F0/F1/F2/F3/F4 5/7/13/8/8	F0/F1/F2/F3/F4 3/5/5/5/3	Independent test set	≥ F3	0.750	0.923	6	1	12	2
Piscaglia, 2006 [37]	Spain	Retrospective	Chronic hepatitis C	ANN	Clinical data	F0/F1/F3/F4 216/176/87/31	F3/total 23/96	Independent test set	≥ F3	0.783	0.890	18	8	65	5
Wang, 2010 [36]	China	Retrospective	Chronic hepatitis C	ANN	Clinical data	F0-1/F2-4 166/60	F0-1/F2-4 80/36	Independent test set	≥ F2	0.917	0.800	33	16	64	3
Raoufy, 2011 [39]	Iran	Prospective	Chronic hepatitis B	ANN	Clinical data	Cirrhotic/non-cirrhotic 11/75	Cirrhotic/non-cirrhotic 8/50	Independent test set	F4	0.875	0.920	7	4	46	1
Pournik, 2014 [35]	Iran	Retrospective	NAFLD patients	ANN	Clinical data	Cirrhotic/non-cirrhotic 52/248	Cirrhotic/non-cirrhotic 15/65	Independent test set	F4	0.66	0.99	44	4	309	23
Shousha, 2018 [34]	Egypt	Retrospective	Chronic hepatitis C	ANN^a^, DT	Clinical data	F0-2/F3-4 204/223	N/A	k-fold cross validation	≥ F3	0.825	0.811	184	39	165	39
Wei, 2018 [33]	USA	Retrospective	Chronic hepatitis B, C	DT, RF, GB^a^	Clinical data	S0/S1/S2/S3/S4 46/169/134/56/85	S0/S1/S2/S3/S4 15/21/12/11/27	Independent test set	S4	0.78	0.85	21	9	50	6
									≥ S3	0.84	0.85	32	7	41	6
Li, 2019 [38]	China	Retrospective	Chronic hepatitis B	DT^a^, RF^a^, LR, SVM	Clinical data	460 patients	460 patients	Independent test set	F4	0.596	0.705	56	108	258	38
									≥ F3	0.939	0.803	176	54	219	11
									≥ F2	0.970	0.763	319	31	100	10
Kuppili, 2017 [26]	Portugal	Prospective	Mixed population	ELM	USG	NAFLD/non-NAFLD 36/27 patients	N/A	K-fold cross validation		0.913	0.921	33	2	25	3
Byra, 2018 [24]	Poland	Prospective	Obese population	CNN	USG	NAFLD/non-NAFLD 38/17 patients	N/A	Leave one out cross validation		1.000	0.882	38	2	15	0

*ANN* artificial neural networks, *CNN* convolutional neural networks, *NB* Naïve Bayes, *RF* random forest, *KNN* k-nearest neighbor, *SVM* support vector machine, *MLP* multilayer perception, *DT* decision tree, *GB* gradient boosting, *LR* logistic regression, *ELM* extreme learning machine, *F4* diagnosis of cirrhosis, *≥ F3* diagnosis of advanced fibrosis (F3–F4), *≥ F2* diagnosis of significant fibrosis (F2–F4)

^a^Selected AIs in the analysis

### Overall performance of AI in the diagnosis of liver cirrhosis

**Fig. 2:**
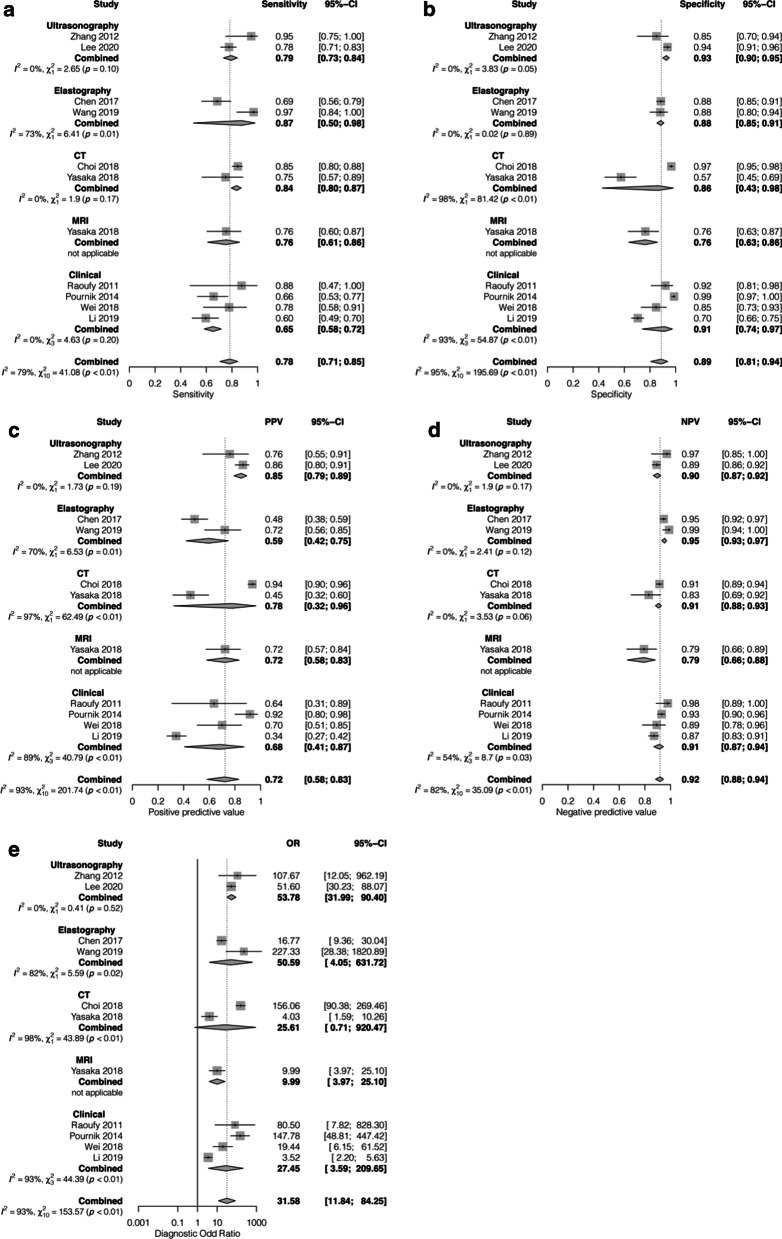
Sensitivity (**a**), specificity (**b**), positive predictive value (**c**), negative predictive value (**d**) and diagnostic odds ratio (**e**) of AI-assisted diagnosis of liver cirrhosis (F4) with subgroup analysis according to diagnostic modality (ultrasonography, elastography, computed tomography and clinical data)

First, we focused on the performance of AI in diagnosing liver cirrhosis (METAVIR F4). A total of 11 studies were included in this analysis [22, 25, 27–30, 32, 33, 35, 38, 39]. Five studies developed AI models using CNNs [22, 28–30, 32], 3 used ANNs [25, 35, 39], and the other 3 studies developed multiple AI models [27, 33, 38]. Different imaging modalities were also employed as inputs for the AI systems: ultrasound was used in 2 studies [22, 25], elastography in 2 studies [27, 28], CT in 2 studies [29, 30], and MRI in 1 study [32]; 4 studies used multiple clinical and laboratory parameters as AI inputs [33, 35, 38, 39]. The results of the meta-analysis showed that AI-assisted systems were able to diagnose cirrhosis with a pooled sensitivity, specificity, PPV, and NPV of 0.78 (95% CI: 0.71–0.85), 0.89 (95% CI: 0.81–0.94), 0.72 (95% CI: 0.58–0.83) and 0.92 (95% CI: 0.88–0.94), respectively. The pooled DOR was 31.58 (95% CI: 11.84–84.25) (Fig. 2). For the sensitivity-focused analysis of the 11 studies, there was no change in the pooled sensitivity. On the other hand, the pooled specificity increased to 0.94 (95% CI: 0.86–0.97) in the specificity-focused analysis (Additional file 1: Table S2).

### Overall performance of AI in the diagnosis of advanced fibrosis (METAVIR ≥ F3) and significant fibrosis (METAVIR ≥ F2)

We identified 10 studies using AI models to diagnose advance fibrosis (≥ F3) [27–30, 32–34, 37, 38, 40]. Four studies developed CNNs [28–30, 32], 1 study developed an ANN [37], 1 study utilized SVM [40], and the other 4 studies developed multiple AI models [27, 33, 34, 38]. The AI models were integrated into elastrography in 2 studies [27, 28], CT images in 2 studies [29, 30], MRI images in 2 study [32, 40] and clinical and laboratory parameters in the other 4 studies [33, 34, 37, 38]. After combining all studies, AI-assisted analysis systems had a pooled sensitivity, specificity, PPV and NPV of 0.86 (95% CI 0.80–0.90), 0.87 (95% CI 0.80–0.92), 0.85 (95% CI 0.75–0.91), and 0.88 (95% CI 0.82–0.92), respectively, and a DOR of 37.79 (95% CI 16.01–89.19) for the diagnosis of advanced fibrosis. Sensitivity and specificity-focused analysis found similar pooled sensitivity but increased pooled specificity to 0.89 (95% CI 0.81–0.93). (Additional file 1: Table S2).

There were 8 studies investigating the performance of AI-assisted systems for the diagnosis of significant fibrosis (≥ F2) [22, 23, 27, 28, 30, 32, 36, 38]. Four studies used CNNs as AI models [23, 28, 29, 31], 1 study utilized an ANN [36], and the other 3 studies used multiple AI models [23, 27, 38]. In this group, the AI models were integrated into ultrasonography in 2 studies [22, 23], elastography in 2 studies [27, 28], CT in 1 study [30], MRI in 1 study [32], and clinical and laboratory parameters in 2 studies [36, 38]. We found that the pooled sensitivity, specificity, PPV and NPV were 0.86 (95% CI 0.78–0.92), 0.81 (95% CI 0.77–0.84), 0.88 (95% CI 0.80–0.93) and 0.77 (95% CI 0.58–0.89), respectively, and the DOR was 26.79 (95% CI 14.47–49.62). In the sensitivity-focused analysis, the pooled sensitivity increased to 0.91 (95% CI 0.76–0.97) while the specificity remained the same in specificity-focused analysis. (Additional file 1: Table S2).

### Subgroup analysis by diagnostic modality

We observed substantial heterogeneity in the overall performance of AI-assisted diagnosis system, e.g., *I*^2^ was 79%, 95%, 93%, 82% and 93% for the pooled sensitivity, specificity, PPV, NPV and DOR, respectively, for the diagnosis of liver cirrhosis. We conducted additional subgroup analyses by diagnostic modality for each stage of fibrosis (Table 2). As expected, there were statistically significant differences in the pooled sensitivity, specificity, PPV, NPV and DOR among different diagnostic modalities. In most subgroups, the *I*^2^ values were markedly decreased.

**Table 2:** Sensitivity, specificity, positive predictive value, negative predictive value and diagnostic odds ratio of AI-assisted diagnosis of significant liver fibrosis (F2–4), advanced fibrosis (F3–4) and cirrhosis (F4) with subgroup analysis according to diagnostic modality (ultrasonography, elastography, computed tomography, clinical data) and population (at-risk population, general population)

Analysis	No. of studies	Pooled sensitivity (95%-CI)	I^2^ (%)	Pooled specificity (95%-CI)	I^2^ (%)	Pooled positive predictive value (95%-CI)	I^2^ (%)	Pooled negative predictive value (95%-CI)	I^2^ (%)	Pooled diagnostic odd ratio (95%-CI)	I^2^ (%)	AUC
*Cirrhosis (F4)*												
Overall	11	0.78 (0.71–0.85)	79^a^	0.89 (0.81–0.94)	95^a^	0.72 (0.58–0.83)	93^a^	0.92 (0.88–0.94)	82^a^	31.58 (11.84–84.25)	93^a^	0.85
Subgroup: modality												
Ultrasonography	2	0.79 (0.73–0.84)	0	0.93 (0.90–0.95)	0	0.85 (0.79–0.89)	0	0.90 (0.87–0.92)	0	53.78 (31.99–90.40)	0	0.95
Elastography	2	0.87 (0.50–0.98)	73^a^	0.88 (0.85–0.91)	0	0.59 (0.42–0.75)	70^a^	0.95 (0.93–0.97)	0	50.59 (4.05–631.72)	82^a^	0.89
CT	2	0.84 (0.80–0.87)	0	0.86 (0.43–0.98)	98^a^	0.78 (0.32–0.96)	97^a^	0.91 (0.88–0.93)	0^a^	25.61 (0.71–920.47)	98^a^	0.84
MRI	1	0.76 (0.61–0.86)	–	0.76 (0.63–0.86)	–	0.72 (0.58–0.83)	–	0.79 (0.66–0.88)	–	9.99 (3.79–25.10)	–	–
Clinical data	4	0.65 (0.58–0.72)	0	0.91 (0.74–0.97)	93^a^	0.68 (0.41–0.87)	89^a^	0.91 (0.87–0.94)	54^a^	27.45 (3.59–209.65)	93^a^	0.68
Subgroup difference, Q		25.02 (*p* < 0.01)^b^		14.95 (*p* < 0.01)^b^		13.11 (*p* = 0.01)^b^		20.57 (*p* < 0.01)^b^		9.90 (*p* = 0.04)^b^		
Subgroup: population												
At-risk population	7	0.80 (0.65–0.90)	84^a^	0.90 (0.80–0.95)	93^a^	0.67 (0.50–0.80)	89^a^	0.94 (0.90–0.97)	75^a^	36.78 (10.67–126.84)	90^a^	0.89
General population	4	0.80 (0.75–0.85)	34	0.87 (0.67–0.96)	96^a^	0.79 (0.57–0.92)	94^a^	0.88 (0.83–0.92)	60^a^	24.89 (5.57–111.16)	95^a^	0.83
Subgroup difference, Q		0.00 (*p* = 0.99)		0.12 (*p* = 0.73)		1.01 (*p* = 0.32)		4.57 (*p* = 0.03)^b^		0.16 (*p* = 0.69)		
*Advanced fibrosis (F3–4)*												
Overall	10	0.86 (0.80–0.90)	80^a^	0.87 (0.80–0.92)	89^a^	0.85 (0.75–0.91)	92^a^	0.88 (0.82–0.92)	84^a^	37.79 (16.01–89.19)	91^a^	0.92
Subgroup: modality												
Elastography	2	0.84 (0.74–0.91)	53^a^	0.94 (0.77–0.99)	59^a^	0.92 (0.49–0.99)	84^a^	0.91 (0.88–0.94)	0	98.96 (5.06–1936.46)	87^a^	0.93
CT	2	0.89 (0.70–0.96)	90^a^	0.87 (0.55–0.97)	95^a^	0.88 (0.55–0.98)	96^a^	0.87 (0.65–0.95)	88^a^	45.86 (0.78–2698.17)	98^a^	0.93
MRI	2	0.78 (0.67–0.86)	0	0.79 (0.65–0.88)	0	0.85 (0.74–0.92)	0	0.70 (0.57–0.81)	0	12.21 (4.96–30.08)	0	0.83
Clinical data	4	0.87 (0.79–0.92)	69^a^	0.82 (0.79–0.92)	0	0.79 (0.75–0.83)	8	0.90 (0.83–0.95)	77^a^	32.81 (17.05–63.15)	61^a^	0.86
Subgroup difference, Q		2.89 (*p* = 0.40)		2.82 (*p* = 0.42)		2.43 (*p* = 0.49)		18.76 (*p* < 0.01)^b^		3.99 (*p* = 0.26)		
Subgroup: population												
At-risk population	6	0.86 (0.80–0.91)	71^a^	0.87 (0.81–0.91)	77^a^	0.82 (0.70–0.90)	91^a^	0.90 (0.86–0.94)	73^a^	36.70 (20.49–65.75)	67^a^	0.90
General population	4	0.85 (0.71–0.93)	84^a^	0.86 (0.67–0.94)	88^a^	0.88 (0.71–0.95)	92^a^	0.83 (0.67–0.92)	84^a^	29.78 (2.63–336.82)	96^a^	0.90
Subgroup difference, Q		0.05 (*p* = 0.82)		0.03 (*p* = 0.87)		0.41 (*p* = 0.52)		1.94 (*p* = 0.16)		0.03 (*p* = 0.87)		
*Significant fibrosis (F2–4)*												
Overall	8	0.86 (0.78–0.92)	91^a^	0.81 (0.77–0.84)	39^a^	0.88 (0.80–0.93)	90^a^	0.77 (0.58–0.89)	95^a^	26.79 (14.47–49.62)	77^a^	0.86
Subgroup: Modality												
Ultrasonography	2	0.90 (0.87–0.93)	0	0.82 (0.77–0.85)	0	0.83 (0.79–0.87)	0	0.86 (0.72–0.94)	80^a^	37.53 (18.66–75.49)	48	0.92
Elastography	2	0.73 (0.68–0.77)	0	0.87 (0.82–0.90)	0	0.92 (0.71–0.98)	79^a^	0.62 (0.29–0.86)	95^a^	18.84 (12.24–29.00)	0	0.85
CT	1	0.76 (0.64–0.85)	–	0.68 (0.52–0.81)	–	0.80 (0.68–0.88)	–	0.63 (0.48–0.77)	–	6.79 (2.77–16.66)		–
MRI	1	0.84 (0.75–0.91)	–	0.70 (0.38–0.90)	–	0.96 (0.89–0.99)	–	0.33 (0.17–0.55)	–	12.67 (2.92–54.96)		–
Clinical data	2	0.96 (0.94–0.98)	0	0.78 (0.72–0.83)	0	0.83 (0.61–0.94)	90^a^	0.93 (0.88–0.96)	0	80.11 (37.50–171.16)	19	0.86
Subgroup difference, Q		76.00 (*p* < 0.01)^b^		12.07 (*p* = 0.02)^b^		9.08 (*p* = 0.06)		45.04 (*p* < 0.01)^b^		20.79 (*p* < 0.01)^b^		
Subgroup: population												
At-risk population	5	0.87 (0.74–0.94)	93^a^	0.82 (0.77–0.86)	39^a^	0.88 (0.77–0.94)	90^a^	0.81 (0.60–0.93)	94^a^	33.99 (15.73–73.41)	74^a^	0.88
General population	3	0.86 (0.77–0.91)	72^a^	0.78 (0.77–0.87)	32^a^	0.87 (0.74–0.94)	82^a^	0.69 (0.34–0.91)	94^a^	17.06 (4.06–71.69)	87^a^	0.87
Subgroup difference, Q		0.07 (*p* = 0.79)		0.43 (*p* = 0.51)		0.00 (*p* = 0.95)		0.51 (*p* = 0.47)		0.69 (*p* = 0.41)		

^a^*p* value for Cochrane Q < 0.1

^b^Significant difference between subgroups

For the diagnosis of cirrhosis, the pooled sensitivity, specificity, PPV, NPV and DOR of different diagnostic modalities were significantly different. The sensitivities were 0.79 (95% CI 0.73–0.84), 0.87 (95% CI 0.50–0.98), 0.84 (95% CI 0.80–0.87), and 0.65 (95% CI 0.58–0.72), and the specificities were 0.93 (95% CI 0.90–0.95), 0.88 (95% CI 0.85–0.91), 0.86 (95% CI 0.43–0.98) and 0.91 (95% CI 0.74–0.97), for ultrasonography, elastrography, CT, and clinical and laboratory parameters, respectively (*p* < 0.01 both). Significant differences in the PPV, NPV and DOR among AI-assisted systems for the diagnosis of cirrhosis were also found (*p* = 0.01, < 0.01 and 0.04, respectively) (Table 2). In the subgroup analyses, the heterogeneity of most diagnostic subgroups of cirrhosis was markedly reduced. For example, *I*^2^ of the ultrasonography subgroup was 0% for the pooled sensitivity, specificity, PPV, NPV and DOR. Similarly, *I*^2^ was 0% for the pooled specificity and NPV of the elastrography subgroup, 0% for the pooled sensitivity and NPV of the CT subgroup and 0% for the pooled sensitivity of the clinical parameters subgroup (Table 2, Fig. 2).

For advanced liver fibrosis (≥ F3), we observed a smaller magnitude of differences in diagnostic performance among diagnostic subgroups, with a smaller reduction in *I*^2^ values after subgroup analyses than the subgroups of cirrhosis. For instance, a statistically significant difference was only detected in the pooled NPV among diagnostic subgroups (*p* < 0.01) (Table 2, Additional file 1: Fig. S1).

The results of the subgroup analyses of significant liver fibrosis (F2-4) stage were similar to those of cirrhosis, i.e., there were significant differences in the pooled sensitivity, specificity, NPV and DOR among diagnostic modality groups (*p* < 0.05), and the heterogeneity accessed by *I*^*2*^ was greatly reduced in several subgroups. The *I*^2^ values were 0% for the pooled sensitivity, specificity and PPV in the ultrasonography subgroup, 0% for the pooled sensitivity, specificity and DOR in the elastography subgroup, and 0% for the pooled sensitivity, specificity and NPV in the clinical data subgroup (Table 2, Additional file 1: Fig. S2).

**Fig. 3:**
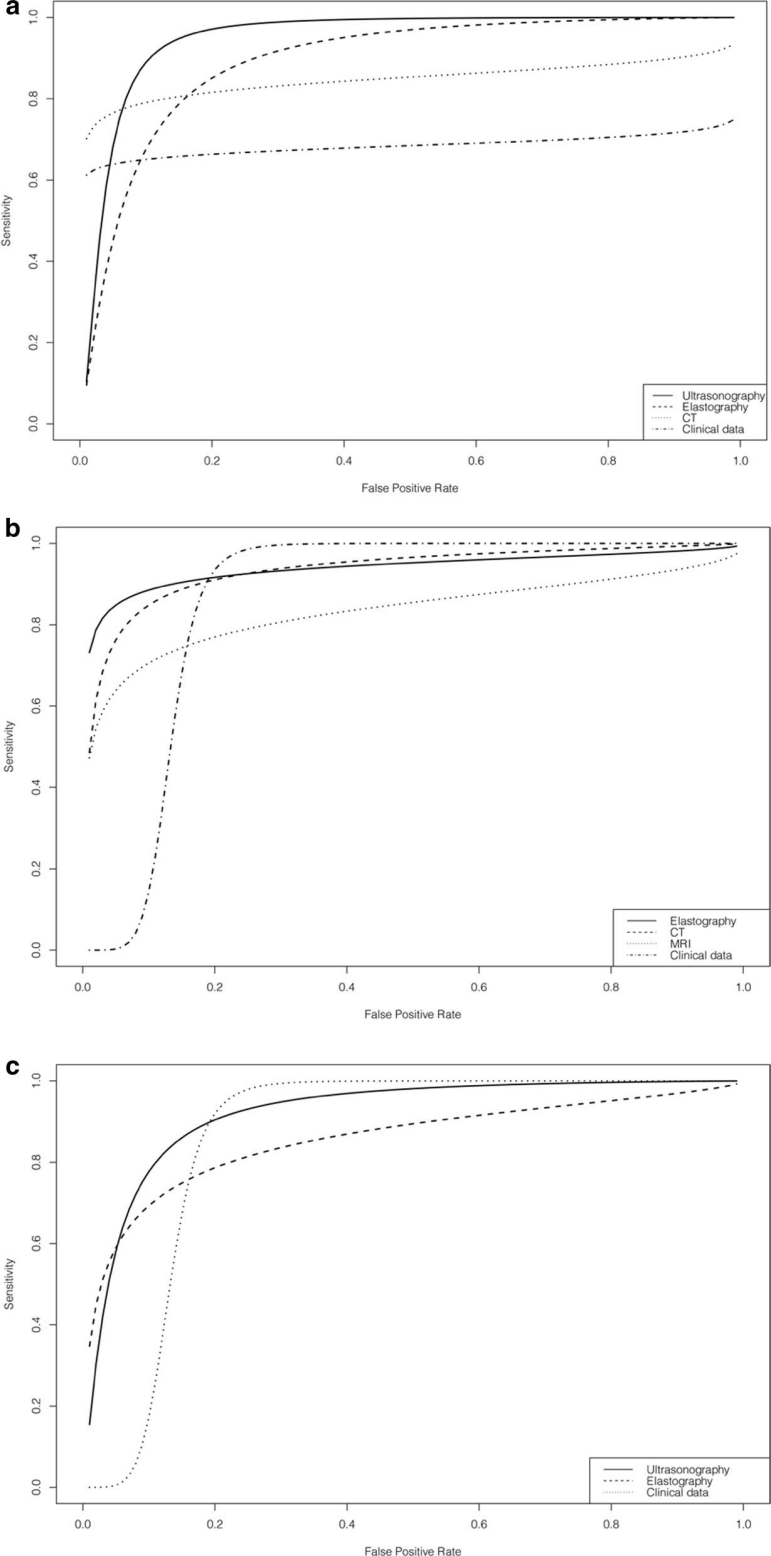
SROC curves demonstrating performance of AI-assisted diagnosis of liver cirrhosis (F4) (**a**), advanced fibrosis (F3–4) (**b**) and significant liver fibrosis (F2–4) (**c**) with subgroup analysis according to diagnostic modality (ultrasonography, elastography, computed tomography and clinical data)

Figure 3 shows the SROC curves of AI-assisted systems for the diagnosis of cirrhosis, advanced fibrosis and significant fibrosis with subgroup analysis by diagnostic modality. The overall AUC values were 0.85, 0.92 and 0.86 for the diagnosis of cirrhosis, advanced fibrosis and significant fibrosis, respectively. AUC values of subgroup analyses of different diagnostic modalities are shown in Table 2.

### Subgroup analysis by study population

We were able to identify 2 population groups in the selected studies. The first group of studies was conducted in a general population without any specific liver disease, while the second group was conducted in an “at-risk” population of individuals who already suffered from chronic liver diseases such as chronic viral hepatitis B and C infections. Therefore, we performed subgroup analyses according to the study population, i.e., the at-risk population and general population. The performance of AI-assisted systems for the diagnosis of F2-F4 fibrosis is summarized in Table 2. In contrast to the aforementioned subgroup analysis, the sensitivity and specificity of AI-assisted diagnostic systems in the at-risk population were similar to those in the general population in all stages of liver fibrosis. The heterogeneity was not dramatically reduced, and the subgroups’ *I*^*2*^ values remained high (70–90%). Additionally, there were no significant differences in diagnostic performance between subgroups (*p* ≥ 0.05) in almost all stages of liver fibrosis. Therefore, we could infer that different populations are unlikely to have an impact on the performance of AI-assisted systems for diagnosing liver fibrosis. To confirm this finding, we further performed a meta-regression analysis with population as a covariate. The mixed effects model showed no statistically significant results, with *p* = 0.69, 0.70 and 0.35 for F4, ≥ F3 and ≥ F2 stages, respectively.

### Subgroup analysis by AI classifiers

We divided AI-classifiers of the included studies into 2 main subgroups, i.e., neural network and non-neural network. Performance of each subgroup is shown in Additional file 1: Table S3. We found that the performance of the 2 subgroups were relatively similar except for a slightly better sensitivity, specificity, PPV and DOR in the neural network group for the diagnosis of cirrhosis. There was no significant difference between AI-classifier subgroups, except for the pooled sensitivity and PPV for the diagnosis of cirrhosis as well as pooled NPV for the diagnosis of advanced fibrosis. We further stratified neural network-assisted studies by diagnostic modalities (ultrasonography, elastography, CT, MRI and clinical data) as well as population (at-risk, general population) (Additional file 1: Table S4). Furthermore, there was a reduction in heterogeneity after subgroup by modalities. For example, *I*^2^ values were 0 for the pooled sensitivity, specificity, PPN, NPV and DOR in the diagnosis of cirrhosis by neural network-assisted ultrasonography and the diagnosis of advanced fibrosis by neural network-assisted clinical parameters. Difference between modalities were also observed in the pooled sensitivity, specificity, NPV and DOR for diagnosing cirrhosis as well as specificity, PPV, NPV and DOR for classifying advanced fibrosis; whereas subgroups by population revealed no significant change in overall performance or heterogeneity.

### Overall performance of AI in the diagnosis of nonalcoholic fatty liver disease (NAFLD)

Only 2 studies on the AI-assisted diagnosis of NAFLD had liver biopsy as the gold standard [24, 26]. One used an ANN, and the other one used a CNN as AI models. The pooled sensitivity, specificity, PPV, NPV and DOR were 0.97 (95% CI 0.76–1.00), 0.91 (95% CI 0.78–0.97), 0.95 (95% CI 0.87–0.98), 0.93 (95% CI 0.80–0.98), and 191.52 (95% CI 38.83–944.81), respectively, with *I*^*2*^ of 0% for all (Additional file 1: Table S5).

### Publication bias

Deeks funnel plots were generated for publication bias assessments. The slope coefficients were relatively symmetrical with P values of 0.30, 0.21 and 0.35 for the diagnosis of cirrhosis, advanced fibrosis and significant fibrosis, respectively (Additional file 1: Fig. S3), suggesting that publication bias was not present.

## Discussion

In this meta-analysis, AI-assisted models had good performance in the assessment of liver fibrosis and steatosis. Interestingly, for the detection of cirrhosis, AI-assisted imaging-based models had greater sensitivities than AI-assisted clinical-based models, i.e., 0.79–0.87 versus 0.65. By contrast, for the diagnosis of significant fibrosis, clinical-based models had a greater sensitivity (0.96 versus 0.73–0.90) but less specificity (0.78 versus 0.82–0.87) than imaging-based models. The NPV of AI-assisted models for detecting advanced liver fibrosis and cirrhosis were approximately 90%, implying that the AI-assisted models were able to help guide clinical decisions that the patients unlikely had liver fibrosis, without the need for invasive methods such as liver biopsy.

AI-aided systems have some advantages over conventional noninvasive diagnostic tools. Unlike ultrasonography, which is an operator-dependent modality, AI utilizes multiple features from ultrasonographic images as inputs to systematically analyze the images, thus reducing bias in the image interpretation. Moreover, AI-assisted diagnosis systems can potentially be used in both the general population and at-risk population. This was suggested by the results of the meta-regression analysis with population as a covariate and by the similar performance of AI-assisted systems between the 2 populations.

Transient elastography is currently the most commonly used noninvasive tool for staging liver fibrosis. A recent meta-analysis showed that transient elastography had AUCs of 0.84, 0.89, and 0.94 for the diagnosis of ≥ F2, ≥ F3 and F4 stage fibrosis, respectively [41, 42]. Real-time elastography has also been frequently used as an alternative to transient elastography with an AUC of 0.72, 0.86 and 0.69 for the diagnosis of liver cirrhosis, advanced fibrosis and significant fibrosis, respectively [43]. Our meta-analysis showed that AI-assisted elastography had higher AUCs for the diagnosis of all stages of liver fibrosis than real-time elastography. When comparing to transient elastography, AI-assisted elastography had a slightly lower AUC for identifying liver cirrhosis, but higher AUCs for classifying advanced fibrosis and significant fibrosis. Interestingly, among the 3 AI-assisted systems, AI-assisted ultrasonography had the best performance (Table 3). This could possibly be due to the difference in types of input data. Studies using AI-assisted ultrasonography incorporated inputs with relatively larger region of interests (ROIs) and extracted different categories of radiomics, compared to AI-assisted elastography studies. Therefore, AI performance could be affected by the selected inputs. Further studies to specify the most appropriate inputs for each AI classifier is warranted in order to maximize the AI performance. Due to the satisfactory performance of AI-assisted ultrasonography, AI has a potential application for staging liver fibrosis in areas where elastography machines are not available. Likewise, the FIB-4 score and APRI score are the most commonly used clinical parameters for predicting liver fibrosis. We found that, in line with the AI-assisted image analysis model, the AI-assisted clinical-based model had a lower AUC value for the diagnosis of stage F4 fibrosis but higher AUC values for the diagnosis of stage ≥ F2 and ≥ F3 fibrosis. Nevertheless, after excluding one study [35] which had a different specific population, focusing only on cirrhosis in NALFD patients, the AUC value for F4 fibrosis dramatically increased from 0.68 to 0.86 which was better than APRI and FIB-4.

**Table 3:** Sensitivity, specificity and area-under-the-curve (AUC) of AI-assisted ultrasonography, AI-assisted elastography, and AI-assisted clinical data for the diagnosis of liver cirrhosis (F4), advanced fibrosis (F3–4) and significant liver fibrosis (F2–4)

Analysis	AI-assisted ultrasonography	AI-assisted elastography	AI-assisted clinical data	Transient elastography [41, 42]	Real-time elastography [43]	APRI [44]	FIB-4 [44]
Cirrhosis (F4)						(Cut-off 2.0)	(Cut-off 1.62–2.65)
Sensitivity	0.79 (0.73–0.84)	0.87 (0.50–0.98)	0.65 (0.58–0.72)	0.83 (0.79–0.86)	0.74 (0.63–0.82)	0.31 (0.13–0.63)	0.64 (0.39–0.77)
Specificity	0.93 (0.90–0.95)	0.88 (0.85–0.91)	0.91 (0.74–0.97)	0.89 (0.87–0.91)	0.84 (0.79–0.88)	0.89 (0.81–0.96)	0.86 (0.75–0.98)
AUC	0.95	0.89	0.68	0.94	0.72	0.72	0.78
Advanced fibrosis (F3–4)						(Cut-off 0.5)	(Cut-off 1.45)
Sensitivity	–	0.84 (0.74–0.91)	0.87 (0.79–0.92)	0.82 (0.78–0.86)	0.82 (0.75–0.88)	0.73 (0.63–0.83)	0.63 (0.50–0.71)
Specificity	–	0.94 (0.77–0.99)	0.82 (0.79–0.85)	0.86 (0.82–0.89)	0.81 (0.72–0.88)	0.55 (0.37–0.72)	0.56 (0.14–0.80)
AUC	–	0.93	0.86	0.89	0.86	0.76	0.80
Significant fibrosis (F2–4)						(Cut-off 0.5)	(Cut-off 1.45)
Sensitivity	0.90 (0.87–0.93)	0.73 (0.68–0.77)	0.96 (0.94–0.98)	0.79 (0.74–0.82)	0.79 (0.75–0.83)	0.70 (0.35–0.97)	0.65 (0.52–0.87)
Specificity	0.82 (0.77–0.85)	0.87 (0.82–0.90)	0.78 (0.72–0.83)	0.78 (0.72–0.83)	0.76 (0.68–0.82)	60 (0.34–0.87)	0.74 (0.65–0.85)
AUC	0.92	0.85	0.86	0.84	0.69	0.72	0.76

Pooled sensitivity, specificity and AUC of transient elastography, real-time elastography, AST to Platelet Ratio Index (APRI) and Fibrosis-4 (FIB-4) for diagnosis of liver fibrosis from previous meta-analyses [41–44] are also shown

In this meta-analysis, we observed relatively high heterogeneity throughout the study. After performing subgroup analyses categorized by diagnostic modality (ultrasound, elastography, CT, MRI, and clinical data), the heterogeneity was dramatically reduced, i.e., the *I*^2^ value was 0% in many subgroups. Moreover, the performance of most subgroups was significantly different, indicating that the types of diagnostic modality had an impact on the performance of AI models. Interestingly, we found that AI-integrated ultrasonography had exceptional performance with a relatively low heterogeneity throughout the analyses. Because ultrasound machines are widely available, this finding suggests that AI-assisted ultrasonography has tremendous potential for being utilized in real clinical practice.

This is one of the very first meta-analyses of the AI-supported systems in diagnosis of liver diseases. Apart from publications in medical journals, we also included articles from computer science and engineering journals, resulting in a comprehensive review of AI advancements regarding this topic. To reduce the chance of overestimating the diagnostic performance of AI models, only studies that had a validation cohort or equivalent method for evaluating the performance of the developed AI models were included.

There are some limitations in this review and meta-analysis. First of all, there are several imaging modalities and AI classifiers included in the meta-analysis which contributed to the heterogeneity of the overall analysis. For different AI-assisted imaging modalities, we prespecified subgroup analysis by modalities. We also further performed subgroup analysis according to AI classifier, i.e., neural networks and non-neural networks (Additional file 1: Table S3). We observed relatively similar performance except for a relatively better performance in the diagnosis of cirrhosis in the neural networks group. Additionally, we performed another subgroup analysis of imaging modalities and population including only studies with neural network AI classifier (Additional file 1: Table S4). We found that the heterogeneity was decreased. However, it is important to note that the input modalities and AI-assisted systems were not completely identical among studies included in the analysis, interpretation of the pooled diagnostic performance needs to be done with caution. Although there were an acceptable number of studies for meta-analysis, the number of studies of each diagnostic tool was relatively small, given that several modalities are currently used for the assessment of liver fibrosis and steatosis. Therefore, the results of the subgroup analyses of each diagnostic modality need to be interpreted with caution. Furthermore, we selected only studies in which liver biopsy was used as the reference standard; consequently, some studies that demonstrated promising results but did not have liver biopsy to confirm the stage of liver fibrosis or steatosis were excluded. Nine of the 19 studies (47%) were prospective; however, none of the included studies were randomized controlled trials. Only 1 study compared the performance between AI and humans [29]. Interestingly, this study showed that the AI-aided system outperformed humans in staging liver fibrosis in CT images. Most included studies evaluated the performance of the developed AI systems on “internal” validation cohorts, of which the baseline patient characteristics were quite similar to those of the development cohort. Whether these developed AI models can be generalized to other populations in clinical practice needs to be further investigated. Moreover, long-term assessment of AI performance in real clinical settings and studies with direct comparisons between AI and conventional diagnostic methods would be beneficial in investigating real-world positive and negative impacts of the AI-assisted system.

## Conclusions

This meta-analysis demonstrates the promising potential of AI systems for aiding the diagnosis and staging of liver fibrosis and NAFLD. Integrating AI into conventional noninvasive tools yields effective diagnostic tools with an optimal balance of sensitivity and specificity. Validation of these AI models in other independent cohorts is warranted before implementing these AI-assisted systems into clinical practice.

## Supplementary Information

**Additional file 1:** Sensitivity, specificity, PPV, NPV and DOR of AI-assisted diagnosis of advanced fibrosis (F3-4), significant fibrosis (F2-4) and non-alcoholic fatty liver disease, Deeks funnel plot, quality assessment (QUADAS-2), sensitivity-focused and specificity-focused analyses, subgroup analysis according to AI classifiers, search strategy.

## Data Availability

Materials described in the manuscript, including all relevant raw data, will be freely available to any researcher wishing to use them for non-commercial purposes. Please contact Dr. Roongruedee Chaiteerakij, the corresponding author, for any inquiries for the data.

## Abbreviations

AI: Artificial intelligence
TP: True positive
FP: False positive
TN: True negative
FN: False negative
PPV: Positive predictive value
NPV: Negative predictive value
DOR: Diagnostic odd ratio
NAFLD: Non-alcoholic fatty liver disease
NASH: Non-alcoholic steatohepatitis
HBV: Hepatitis B virus
HCV: Hepatitis C virus
CT: Computed tomography
MRI: Magnetic resonance imaging
