# Supplementary material for: Application of artificial intelligence in chronic liver diseases: a systematic review and meta-analysis
Source: BMC Gastroenterol. 2021 Jan 6;21:10. doi: 10.1186/s12876-020-01585-5 (PMC7788739; doi:10.1186/s12876-020-01585-5)
Supplement: Supplementary file 1 — Additional file 1: Sensitivity, specificity, PPV, NPV and DOR of AI-assisted diagnosis of advanced fibrosis (F3-4), significant fibrosis (F2-4) and non-alcoholic fatty liver disease, Deeks funnel plot, quality assessment (QUADAS-2), sensitivity-focused and specificity-focused analyses, subgroup analysis according to AI classifiers, search strategy. [file 12876_2020_1585_MOESM1_ESM.docx]

**Figure S1:** Sensitivity (1A), specificity (1B), positive predictive value (1C), negative predictive value (1D) and diagnostic odds ratio (1E) of AI-assisted diagnosis of advanced fibrosis
(F3-4) with subgroup analysis according to diagnostic modality (ultrasonography, elastography, computed tomography and clinical data)


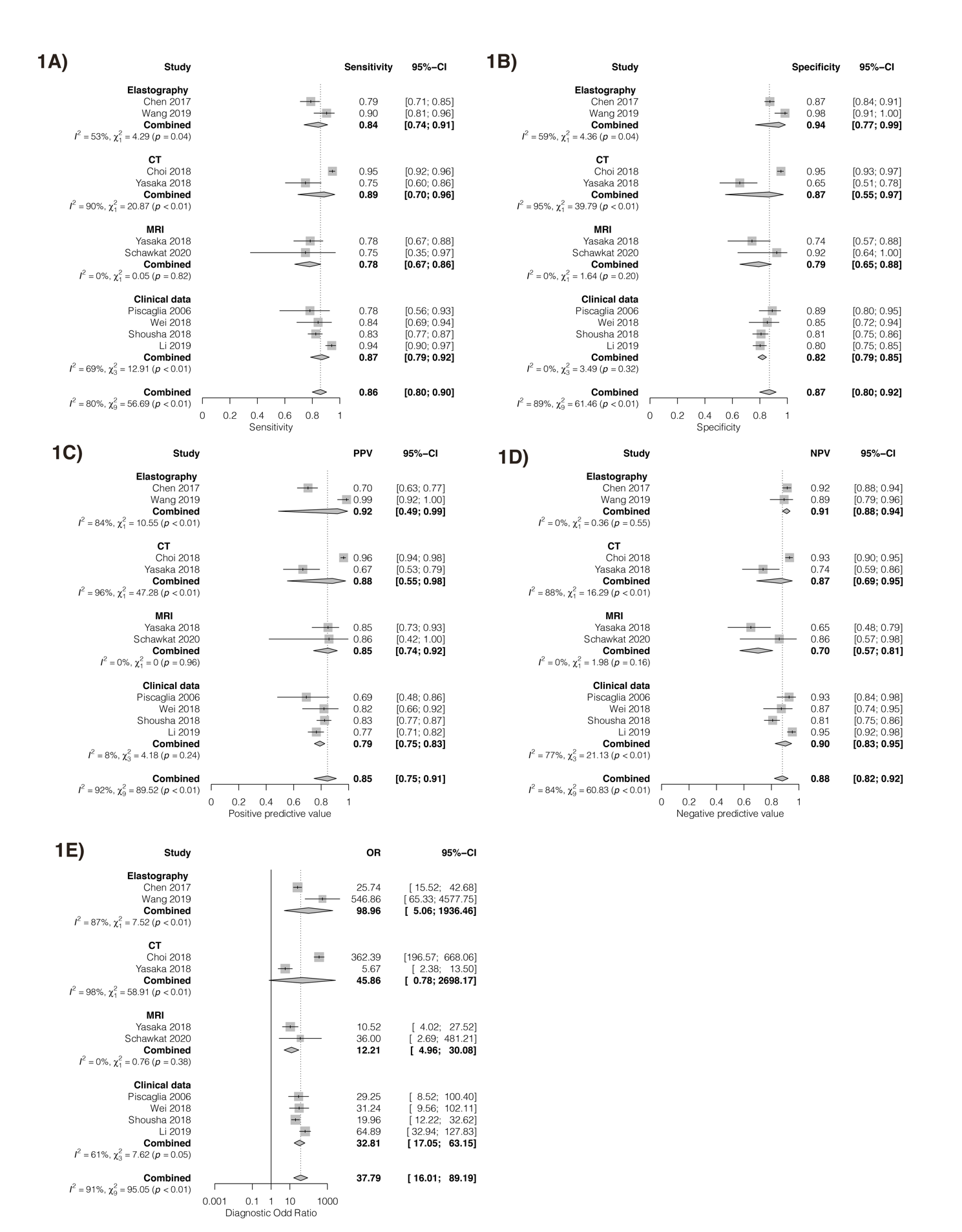


**Figure S2:** Sensitivity (2A), specificity (2B), positive predictive value (2C), negative predictive value (2D) and diagnostic odds ratio (2E) of AI-assisted diagnosis of significant fibrosis
(F2-4) with subgroup analysis according to diagnostic modality (ultrasonography, elastography, computed tomography and clinical data)


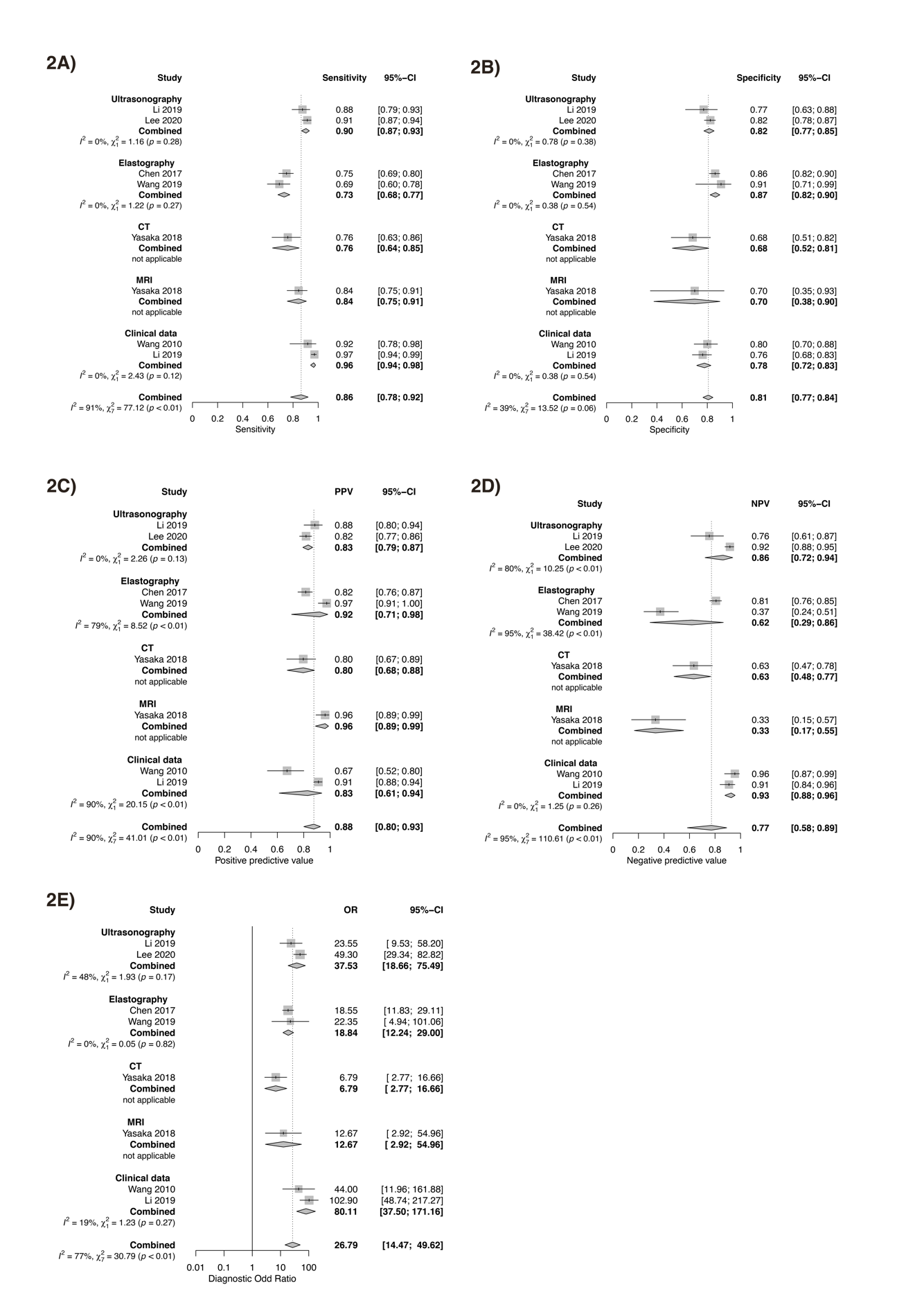


**Figure S3:** Deeks funnel plot of AI-assisted diagnosis of liver cirrhosis (3A), advanced fibrosis (3B), significant fibrosis (3C)


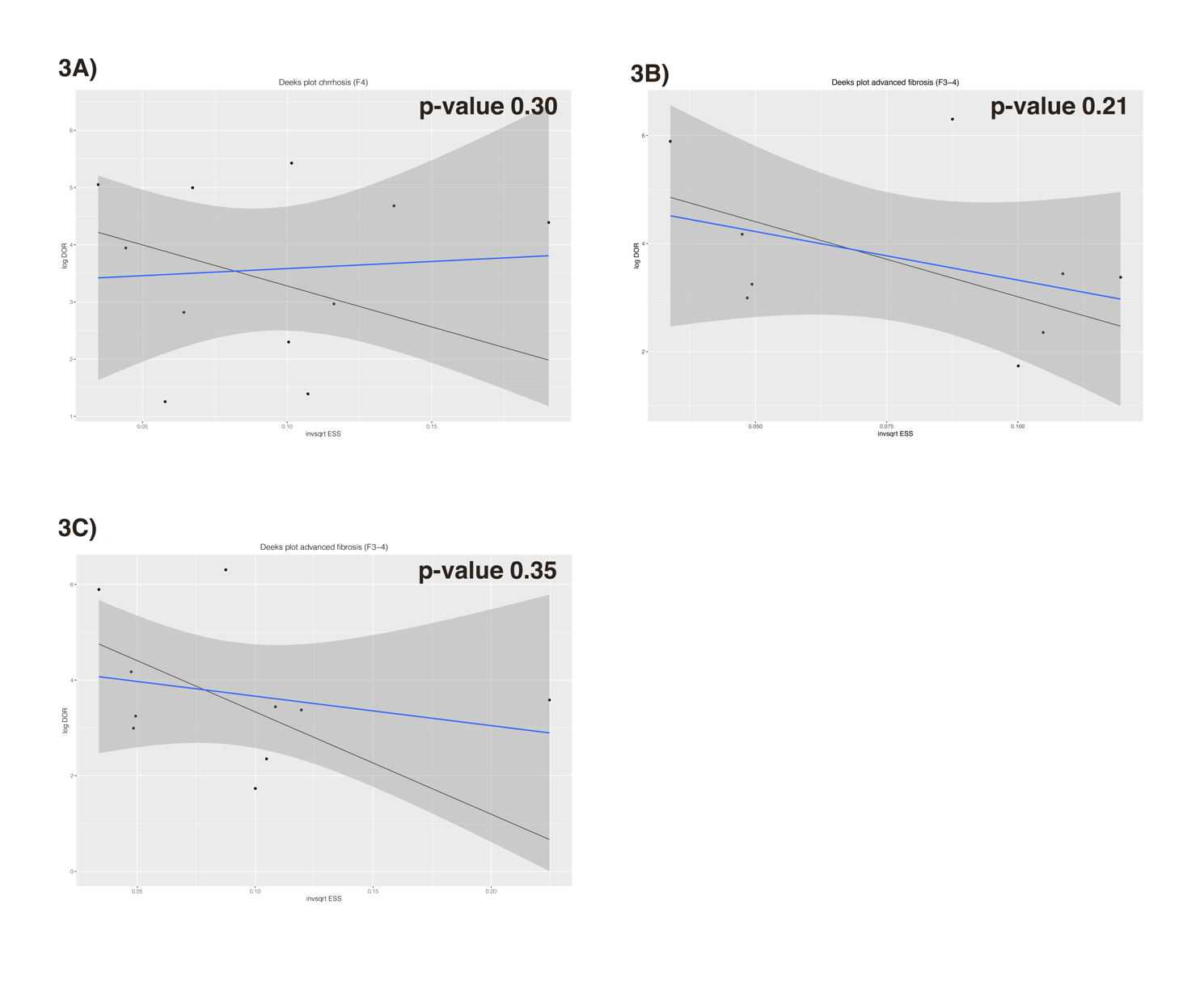


**Table S1:** Quality assessment of included studies using QUADAS-2

| **Reference/year** | **Risk of bias** | | | | **Applicability concerns** | | |
| --- | --- | --- | --- | --- | --- | --- | --- |
|  | **Patient selection (1)** | **Index test (2)** | **Reference standard (3)** | **Flow and timing (4)** | **Patient selection (5)** | **Index test (6)** | **Reference standard (7)** |
| Zhang, 2012^(25)^ | HR | LR | LR | LR | LR | LR | LR |
| Chen, 2017^(27)^ | LR | HR | LR | LR | LR | LR | LR |
| Choi, 2018^(29)^ | LR | LR | LR | LR | LR | LR | LR |
| Yasaka, 2018^(30)^ | UR | LR | LR | LR | LR | LR | LR |
| Yasaka, 2018^(32)^ | LR | LR | LR | LR | LR | LR | LR |
| Li, 2019^(23)^ | LR | HR | LR | LR | LR | LR | LR |
| Wang, 2019^(28)^ | LR | LR | LR | LR | LR | LR | LR |
| Ahmed, 2020^(31)^ | LR | LR | LR | LR | LR | LR | LR |
| Lee, 2020^(22)^ | LR | LR | LR | LR | LR | LR | LR |
| Schawkat, 2020^(40)^ | LR | LR | LR | LR | LR | LR | LR |
| Piscaglia, 2006^(37)^ | LR | LR | LR | LR | LR | LR | LR |
| Wang, 2010^(36)^ | LR | LR | LR | LR | LR | LR | LR |
| Raoufy, 2011^(40)^ | LR | LR | LR | LR | LR | LR | LR |
| Pournik, 2014^(35)^ | LR | HR | UR | LR | LR | LR | LR |
| Shousha, 2018^(34)^ | LR | HR | LR | LR | LR | LR | LR |
| Wei, 2018^(33)^ | LR | UR | UR | LR | LR | LR | LR |
| Li, 2019^(38)^ | LR | LR | UR | LR | LR | LR | LR |
| Kuppili, 2017^(26)^ | LR | HR | LR | LR | LR | LR | LR |
| Byra, 2018^(24)^ | LR | LR | LR | LR | LR | LR | LR |

HR: high risk for bias, LR: low risk for bias, UR: uncertain risk for bias

**Table S2:** Sensitivity, specificity, positive predictive value, negative predictive value and diagnostic odds ratio of sensitivity-focused and specificity-focused AI-assisted diagnosis of significant liver fibrosis (F2-4) and advanced fibrosis (F3-4)

| Analysis | Pooled sensitivity  (95%-CI) | I^2^ (%) | Pooled specificity  (95%-CI) | I^2^ (%) | Pooled positive predictive value  (95%-CI) | I^2^ (%) | Pooled negative predictive value  (95%-CI) | I^2^ (%) | Pooled diagnostic odd ratio  (95%-CI) | I^2^ (%) |
| --- | --- | --- | --- | --- | --- | --- | --- | --- | --- | --- |
| Sensitivity-focused |  |  |  |  |  |  |  |  |  |  |
| Cirrhosis (F4) | 0.78 (0.71 – 0.85) | 79^a^ | 0.89 (0.81 – 0.94) | 95^a^ | 0.72 (0.58 – 0.83) | 93^a^ | 0.92 (0.88 – 0.94) | 82^a^ | 31.58 (11.84 – 84.25) | 93^a^ |
| Advanced fibrosis (F3-4) | 0.86 (0.80 – 0.90) | 80^a^ | 0.87 (0.80 – 0.92) | 89^a^ | 0.85 (0.75 – 0.91) | 92^a^ | 0.88 (0.82 – 0.92) | 84^a^ | 37.79 (16.01 – 89.19) | 91^a^ |
| Significant fibrosis (F2-4) | 0.91 (0.76 – 0.97) | 97^a^ | 0.63 (0.25 – 0.90) | 98^a^ | 0.85 (0.76 – 0.91) | 93^a^ | - | - | 22.63 (12.73 – 40.23) | 67^a^ |
| Specificity-focused |  |  |  |  |  |  |  |  |  |  |
| Cirrhosis (F4) | 0.67 (0.29 – 0.91) | 99^a^ | 0.94 (0.86 – 0.97) | 95^a^ | - | - | 0.90 (0.78 – 0.96) | 98^a^ | 41.43 (17.30 – 99.21) | 87^a^ |
| Advanced fibrosis (F3-4) | 0.85 (0.77 – 0.90) | 86^a^ | 0.89 (0.81 – 0.93) | 91^a^ | 0.86 (0.77 – 0.92) | 90^a^ | 0.87 (0.82 – 0.92) | 85^a^ | 40.41 (16.81 –97.12) | 90^a^ |
| Significant fibrosis (F2-4) | 0.86 (0.78 – 0.92) | 91^a^ | 0.81 (0.77 – 0.84) | 39^a^ | 0.88 (0.80 – 0.93) | 90^a^ | 0.77 (0.58 – 0.89) | 95^a^ | 26.79 (14.47 – 49.62) | 77^a^ |

^a^*p* value for Cochrane Q < 0.1

**Table S3:** Sensitivity, specificity, positive predictive value, negative predictive value and diagnostic odds ratio of AI-assisted diagnosis of significant liver fibrosis (F2-4), advanced fibrosis (F3-4) and cirrhosis (F4) with subgroup analysis according to
artificial intelligence classifier (neural networks, non-neural networks)

| Analysis | No. of studies | Pooled sensitivity  (95%-CI) | I^2^ (%) | Pooled specificity  (95%-CI) | I^2^ (%) | Pooled positive predictive value  (95%-CI) | I^2^ (%) | Pooled negative predictive value  (95%-CI) | I^2^ (%) | Pooled diagnostic odd ratio  (95%-CI) | I^2^ (%) |
| --- | --- | --- | --- | --- | --- | --- | --- | --- | --- | --- | --- |
| Cirrhosis (F4) |  |  |  |  |  |  |  |  |  |  |  |
| Overall | 11 | 0.78 (0.71 – 0.85) | 79^a^ | 0.89 (0.81 – 0.94) | 95^a^ | 0.72 (0.58 – 0.83) | 93^a^ | 0.92 (0.88 – 0.94) | 82^a^ | 31.58 (11.84 – 84.25) | 93^a^ |
| *Subgroup: AI classifier* |  |  |  |  |  |  |  |  |  |  |  |
| Neural networks | 8 | 0.82 (0.74 – 0.88) | 74^a^ | 0.91 (0.81 – 0.96) | 93^a^ | 0.79 (0.67 – 0.88) | 88^a^ | 0.93 (0.87 – 0.96) | 87^a^ | 50.16 (17.65 – 142.59) | 89^a^ |
| Non-neural networks | 3 | 0.66 (0.57 – 0.73) | 79^a^ | 0.82 (0.71 – 0.90) | 89^a^ | 0.49 (0.33 – 0.65) | 84^a^ | 0.91 (0.86 – 0.95) | 71^a^ | 9.93 (2.94 – 33.58) | 90^a^ |
| *Subgroup difference, Q* |  | 7.72 (*p* < 0.01)^b^ |  | 2.01 (*p* = 0.16) |  | 8.43 (*p* < 0.01)^b^ |  | 0.19 (*p* = 0.67) |  | 3.91 (*p* = 0.05) |  |
| Advanced fibrosis (F3-4) |  |  |  |  |  |  |  |  |  |  |  |
| Overall | 10 | 0.86 (0.80 – 0.90) | 80^a^ | 0.87 (0.80 – 0.92) | 89^a^ | 0.85 (0.75 – 0.91) | 92^a^ | 0.88 (0.82 – 0.92) | 84^a^ | 37.79 (16.01 – 89.19) | 91^a^ |
| *Subgroup: AI classifier* |  |  |  |  |  |  |  |  |  |  |  |
| Neural networks | 6 | 0.86 (0.78 – 0.91) | 82^a^ | 0.88 (0.75 – 0.94) | 92^a^ | 0.88 (0.74 – 0.95) | 93^a^ | 0.85 (0.76 – 0.91) | 86^a^ | 39.91 (9.24 – 172.35) | 94^a^ |
| Non-neural networks | 4 | 0.86 (0.76 – 0.93) | 71^a^ | 0.85 (0.80 – 0.88) | 40^a^ | 0.75 (0.71 – 0.91) | 0 | 0.92 (0.89 – 0.95) | 20 | 37.04 (21.43 – 64.01) | 35 |
| *Subgroup difference, Q* |  | 0.02 (*p* = 0.89) |  | 0.33 (*p* = 0.57) |  | 3.25 (*p* = 0.07) |  | 4.13 (*p* = 0.04)^b^ |  | 0.01 (*p* = 0.93) |  |
| Significant fibrosis (F2-4) |  |  |  |  |  |  |  |  |  |  |  |
| Overall | 8 | 0.86 (0.78 – 0.92) | 91^a^ | 0.81 (0.77 – 0.84) | 39^a^ | 0.88 (0.80 – 0.93) | 90^a^ | 0.77 (0.58 – 0.89) | 95^a^ | 26.79 (14.47 – 49.62) | 77^a^ |
| *Subgroup: AI classifier* |  |  |  |  |  |  |  |  |  |  |  |
| Neural networks | 5 | 0.84 (0.74 – 0.90) | 81^a^ | 0.81 (0.77 – 0.84) | 0 | 0.88 (0.74 – 0.95) | 91^a^ | 0.72 (0.40 – 0.91) | 95^a^ | 21.65 (8.59 – 54.59) | 75^a^ |
| Non-neural networks | 3 | 0.90 (0.74 – 0.96) | 93^a^ | 0.81 (0.75 – 0.87) | 55^a^ | 0.88 (0.82 – 0.92) | 69^a^ | 0.83 (0.75 – 0.89) | 64^a^ | 35.05 (11.83 – 103.83) | 87^a^ |
| *Subgroup difference, Q* |  | 0.65 (*p* = 0.42) |  | 0.01 (*p* = 0.92) |  | 0.02 (*p* = 0.89) |  | 0.80 (*p* = 0.37) |  | 0.44 (*p* = 0.51) |  |

^a^*p* value for Cochrane Q < 0.1

^b^significant difference between subgroups

**Table S4:** Sensitivity, specificity, positive predictive value, negative predictive value and diagnostic odds ratio of Neural networks-assisted diagnosis of significant liver fibrosis (F2-4), advanced fibrosis (F3-4) and cirrhosis (F4) with subgroup analysis according to diagnostic modality (ultrasonography, elastography, computed tomography, clinical data) and population (at-risk population, general population)

| Analysis | No. of studies | Pooled sensitivity  (95%-CI) | I^2^ (%) | Pooled specificity  (95%-CI) | I^2^ (%) | Pooled positive predictive value  (95%-CI) | I^2^ (%) | Pooled negative predictive value  (95%-CI) | I^2^ (%) | Pooled diagnostic odd ratio  (95%-CI) | I^2^ (%) |
| --- | --- | --- | --- | --- | --- | --- | --- | --- | --- | --- | --- |
| Cirrhosis (F4) |  |  |  |  |  |  |  |  |  |  |  |
| Overall | 8 | 0.82 (0.74 – 0.88) | 74^a^ | 0.91 (0.81 – 0.96) | 93^a^ | 0.79 (0.67 – 0.88) | 88^a^ | 0.93 (0.87 – 0.96) | 87^a^ | 50.16 (17.65 – 142.59) | 89^a^ |
| *Subgroup: Modality* |  |  |  |  |  |  |  |  |  |  |  |
| Ultrasonography | 2 | 0.79 (0.73 – 0.84) | 0 | 0.93 (0.90 – 0.95) | 0 | 0.85 (0.79 – 0.89) | 0 | 0.90 (0.87 – 0.92) | 0 | 53.78 (31.99 – 90.40) | 0 |
| Elastography | 1 | 0.97 (0.81 – 1.00) | - | 0.88 (0.80 – 0.93) | - | 0.72 (0.57 – 0.83) | - | 0.99 (0.92 – 1.00) | - | 227.33 (28.38 – 1820.89) | - |
| CT | 2 | 0.84 (0.80 – 0.87) | 0 | 0.86 (0.43 – 0.98) | 98^a^ | 0.78 (0.32 – 0.96) | 97^a^ | 0.91 (0.88 – 0.93) | 0^a^ | 25.61 (0.71 – 920.47) | 98^a^ |
| MRI | 1 | 0.76 (0.61 – 0.86) | - | 0.76 (0.63 – 0.86) | - | 0.72 (0.58 – 0.83) | - | 0.79 (0.66 – 0.88) | - | 9.99 (3.97 – 25.10) | - |
| Clinical data | 2 | 0.68 (0.57 – 0.78) | 0 | 0.97 (0.90 – 0.99) | 71^a^ | 0.84 (0.58 – 0.95) | 61^a^ | 0.94 (0.91 – 0.96) | 0 | 132.14 (48.58 – 359.37) | 0 |
| *Subgroup difference, Q* |  | 14.28 (*p* < 0.01)^b^ |  | 18.48 (*p* < 0.01)^b^ |  | 6.45 (*p* = 0.17) |  | 16.74 (*p* < 0.01)^b^ |  | 17.53 (*p* = 0.01)^b^ |  |
| *Subgroup: Population* |  |  |  |  |  |  |  |  |  |  |  |
| At-risk population | 4 | 0.90 (0.69 – 0.97) | 66^a^ | 0.94 (0.84 – 0.98) | 82^a^ | 0.79 (0.66 – 0.88) | 49^a^ | 0.97 (0.91 – 0.99) | 37 | 139.95 (60.79 – 322.17) | 0 |
| General population | 4 | 0.80 (0.75 – 0.85) | 34 | 0.87 (0.67 – 0.96) | 96^a^ | 0.79 (0.57 – 0.92) | 94^a^ | 0.88 (0.83 – 0.92) | 60^a^ | 24.89 (5.57 – 111.16) | 95^a^ |
| *Subgroup difference, Q* |  | 1.09 (*p* = 0.30) |  | 0.93 (*p* = 0.33) |  | 0.00 (*p* = 0.97) |  | 5.65 (*p* = 0.02)^b^ |  | 3.90 (*p* = 0.05) |  |
| Advanced fibrosis (F3-4) |  |  |  |  |  |  |  |  |  |  |  |
| Overall | 6 | 0.86 (0.78 – 0.91) | 82^a^ | 0.88 (0.75 – 0.94) | 92^a^ | 0.88 (0.74 – 0.95) | 93^a^ | 0.85 (0.76 – 0.91) | 86^a^ | 39.91 (9.24 – 172.35) | 94^a^ |
| *Subgroup: Modality* |  |  |  |  |  |  |  |  |  |  |  |
| Elastography | 1 | 0.90 (0.81 – 0.95) | - | 0.98 (0.89 – 1.00) | - | 0.99 (0.90 – 1.00) | - | 0.89 (0.79 – 0.95) | - | 546.86 (65.33 – 4577.75) | - |
| CT | 2 | 0.89 (0.70 – 0.96) | 90^a^ | 0.87 (0.55 – 0.97) | 95^a^ | 0.88 (0.55 – 0.98) | 96^a^ | 0.87 (0.65 – 0.95) | 88^a^ | 45.86 (0.78 – 2698.17) | 98^a^ |
| MRI | 1 | 0.78 (0.67 – 0.87) | - | 0.74 (0.58 – 0.86) | - | 0.85 (0.74 – 0.92) | - | 0.65 (0.49 – 0.78) | - | 10.52 (4.02 – 27.52) | - |
| Clinical data | 2 | 0.82 (0.77 – 0.86) | 0 | 0.83 (0.78 – 0.87) | 0 | 0.81 (0.76 – 0.86) | 0 | 0.87 (0.75 – 0.94) | 0 | 21.03 (13.33 – 33.19) | 0 |
| *Subgroup difference, Q* |  | 4.37 (*p* = 0.22) |  | 7.96 (*p* = 0.05) |  | 7.68 (*p* = 0.05) |  | 10.83 (*p* = 0.01)^b^ |  | 11.17 (*p* = 0.01)^b^ |  |
| *Subgroup: Population* |  |  |  |  |  |  |  |  |  |  |  |
| At-risk population | 3 | 0.84 (0.80 – 0.88) | 0 | 0.91 (0.77 – 0.97) | 82^a^ | 0.89 (0.63 – 0.97) | 89^a^ | 0.87 (0.79 – 0.93) | 55^a^ | 48.50 (11.43 – 205.74) | 78^a^ |
| General population | 3 | 0.86 (0.71 – 0.94) | 89^a^ | 0.83 (0.60 – 0.94) | 92^a^ | 0.87 (0.67 – 0.96) | 93^a^ | 0.82 (0.62 – 0.92) | 90^a^ | 28.30 (1.68 – 477.82) | 97^a^ |
| *Subgroup difference, Q* |  | 0.10 (*p* = 0.75) |  | 0.68 (*p* = 0.41) |  | 0.01 (*p* = 0.90) |  | 1.94 (*p* = 0.16) |  | 0.56 (*p* = 0.45) |  |

^a^*p* value for Cochrane Q < 0.1

^b^significant difference between subgroups

**Table S5:** Sensitivity, specificity, positive predictive value, negative predictive value and diagnostic odds ratio of AI-assisted diagnosis of non-alcoholic fatty liver disease

| Analysis | Pooled sensitivity  (95%-CI) | I^2^ (%) | Pooled specificity  (95%-CI) | I^2^ (%) | Pooled positive predictive value  (95%-CI) | I^2^ (%) | Pooled negative predictive value  (95%-CI) | I^2^ (%) | Pooled diagnostic odd ratio  (95%-CI) | I^2^ (%) |
| --- | --- | --- | --- | --- | --- | --- | --- | --- | --- | --- |
| Non-alcoholic fatty liver disease | | | | | | | | | | |
| Combined | 0.97 (0.76 – 1.00) | 41 | 0.91 (0.78 – 0.97) | 0 | 0.95 (0.87 – 0.98) | 0 | 0.93 (0.80 – 0.98) | 0 | 191.52 (38.82 – 944.81) | 0 |

**Search strategy**

**PubMed**

(((((artificial intelligence[Title] OR computer-assisted[Title]) OR computer-aided[Title]) OR neural network[Title]) OR machine learning[Title]) OR deep learning[Title]) AND (liver[Title] OR hepatic[Title]) AND ((((((fibrosis[Title] OR steatosis[Title]) OR fatty[Title]) OR NASH[Title]) OR NAFLD[Title]) OR cirrhosis[Title]) OR parenchyma[Title] OR parenchymal[Title]) AND ("2000/01/01"[PDAT] : "3000"[PDAT]) AND english[Language]

**Scopus**

(TITLE(artificial intelligence) OR TITLE(computer-assisted) OR TITLE(computer-aided) OR TITLE(neural network) OR TITLE(machine learning) OR TITLE(deep learning) ) AND (TITLE(liver OR hepatic)) AND (TITLE(fibrosis OR steatosis OR fatty OR NASH OR nafld OR steatosis OR cirrhosis OR parenchyma OR parenchymal))

**Web of Science**

TI=(artificial intelligence OR computer-assisted OR computer-aided OR neural network OR machine learning OR deep learning) AND TI=(liver OR hepatic) AND TI=(fibrosis OR steatosis OR fatty OR NASH OR NAFLD OR cirrhosis OR parenchyma OR parenchymal)

**Google Scholar**

allintitle: ("artificial intelligence" OR "computer assisted" OR "computer aided" OR "neural network" OR "machine learning" OR "deep learning”) AND (liver OR hepatic) AND (fibrosis OR steatosis OR fatty OR NASH OR NAFLD OR cirrhosis OR parenchyma OR parenchymal)
